# Regional Differences in and Influencing Factors of Animal Epidemic Risk in China

**DOI:** 10.3389/fvets.2020.00520

**Published:** 2020-09-25

**Authors:** Yanling Li, Long Sun, Wei Zhou, Qingsong Su

**Affiliations:** ^1^College of Public Administration and Law, Hunan Agricultural University, Changsha, China; ^2^College of Humanities and Development Studies, China Agricultural University, Beijing, China

**Keywords:** animal epidemic risk, influencing factors, regional difference, Shapley value, epidemic risk index

## Abstract

Based on data from three major pig diseases, this study calculated the animal disease epidemic index of 31 provinces and autonomous regions in mainland China. We adopted the Gini coefficient to investigate the interregional differences in animal disease epidemic risk and used the Shapley value decomposition method to illustrate the contribution of influencing factors. The results showed that the Gini coefficient remains above 0.60, indicating significant interregional differences in mainland China. Animal breeding level, ecological environment, and animal disease prevention and control contribute most to the interregional differences in animal epidemic risk. The results imply that reducing sewage discharge, increasing pig production, and changing the breeding style from free-range to large-scale farming are measures that may help improve disease prevention and control. This study has implications for providing theoretical references for preventing and controlling animal epidemics and for improving public health governance.

## Introduction

With the rapid development of the livestock and poultry farming industry in mainland China, the density of livestock and poultry breeding has increased greatly, and the trade and circulation of livestock and poultry products have accelerated. This is accompanied by the spread of animal epidemics, leading to a series of public health concerns over environmental damage, zoonotic diseases, and public panic (1). Epidemics in animal populations could pose a great threat to human health and food security (2). This study assessed interregional differences in the risk of animal epidemic diseases and their influencing factors, with the aim of providing helpful measures for animal disease prevention and control and for reducing the occurrence of animal epidemic public crises.

Previous studies have investigated animal epidemic risk prevention and control from various perspectives. Gerardo et al. evaluated the influence of different spatial units (i.e., counties) in Uruguay on the epidemic spread of the foot-and-mouth disease virus (3). Le-Thi et al. studied human diarrhea risks caused by exposure to livestock waste (4). Sung et al. investigated the determinants of porcine epidemic diarrhea virus dissemination because of spatial and temporal factors in Taiwan (5). Damien illustrated what the effectiveness of applying big data means for animal health surveillance to improve risk management (6).

Regional animal husbandry and production not only reflect agricultural development in the region but also indicate the changes in epidemic risk caused by such development to a certain extent. Xu et al. showed that the levels of regional animal husbandry and disease prevention and control are crucial determinants of differences in the epidemic risk of animal diseases across regions (7). Gillespie et al. found that the size of the pig population can be a potential risk factor for the incursion and spread of infectious diseases (8).

Wei et al. pointed out that China has invested significant efforts in improving public animal health infrastructure (9). They reviewed China's animal disease control policies and assessed institutional weaknesses, such as administrative failings, including poorly demarcated and inconsistent oversight as well as weak accountability. Dürr et al. and Limon et al. discussed the relationship between epidemic prevention management protocols and regional differences in the epidemic risk of animal diseases (10, 11). They considered that the qualifications of the personnel involved in epidemic prevention do not keep pace with advances in animal husbandry and should thus be improved. Payen et al. indicated that the structure and dynamics of animal trade play an important role in controlling zoonosis (12).

Mori and Yang et al. concluded that the geographical and ecological environment has a marked impact on human and animal health. In particular, the focal point of an epidemic has a significant influence on its spatial and temporal distribution trends (13, 14). Yang et al. integrated biological and statistical models to determine the environmental factors that impact malaria transmission patterns in China (15). Yang et al. subsequently constructed the animal epidemic status on regional epidemics, potential transmission risk, and institutional ability (16).

Few studies have focused on provincial differences and the factors influencing interregional differences in animal epidemic risk in China. In this study, the epidemic index of animal epidemics in different provinces and municipalities was calculated, and the ArcGIS software was used to draw the epidemic risk map. According to this index, the 31 provinces in mainland China were divided into four risk regions: extremely high, high, medium, and low risk. Additionally, the Gini coefficient was used to measure interregional differences in animal epidemic risk across regions. Finally, 21 provinces and three autonomous regions with the highest risk of pig epidemic diseases were selected to explore the factors influencing differences in animal epidemic risk across regions and their contributions to these interregional differences, by applying a panel data model and the Shapley value decomposition method.

## Materials and Methods

### Sample Selection and Epidemic Disease Index Calculation

China is the leading nation in terms of the number of pigs raised, pig stock, and pork output and consumption. Pork has always been the animal product with the highest output and output value in China. Therefore, this is representative of live pigs, as a research target for studying animal epidemic diseases.

According to the statistic from Veterinary Bulletins of China, 29 provinces and autonomous regions in mainland China (all except Beijing and Tibet) experienced pig diseases from 2010 to 2014, and classical swine fever, swine erysipelas, and swine plague are the three pig diseases that occur most frequently. These three diseases are prevalent in different regions of China and can reflect the regional characteristics of animal diseases in China. The Veterinary Bulletin is a government publication founded and edited by the Ministry of Agriculture and Rural Affairs of the People's Republic of China. It reports the occurrence of animal epidemics throughout the 31 provinces, municipalities, and autonomous regions of China. The provincial animal epidemic situation is identified by calculating the animal epidemic indices in different regions as follows (17):

(1) $$\begin{array}{l} E=(N_{i}/\sum N+O_{i}/\sum O+D_{i}/\sum D\\ \;+\;K_{i}/\sum K)/(A_{i}/\sum A_{i}) \end{array}$$

where E is the epidemic index of a disease in a certain region; *N*_*i*_, *O*_*i*_, *D*_*i*_, and, *K*_*i*_ represent the outbreak frequency of an animal epidemic disease, number of diseased animals, number of deaths, and number of animals slaughtered, respectively; ∑*N*, ∑*O*, ∑*D*, and ∑*K* represent the total outbreak frequency of an animal epidemic disease throughout the country, total number of diseased animals, total number of deaths, and total number of animals slaughtered, respectively; and *A*_*i*_ and ∑*A*_*i*_ represent the total number of animals raised in a certain region and throughout the country over the year, respectively.

By calculating and averaging epidemic indices of classical swine fever, swine erysipelas, and swine plague from 2010 to 2014 in the provinces and autonomous regions, the animal epidemic indices in various regions were obtained. These indices reflect the epidemic situations of provincial animal diseases over the past 5 consecutive years (Table 1). Although in all but 11 provinces the skewness coefficients were >1 (Table 1), suggesting that the median may describe the prevalence distribution better than the mean, we preferred to use the mean to capture the influence of certain years in the probability of having an outbreak. In other words, using the median would likely underestimate the risk of an outbreak for situations in which outbreaks were rare, but did occur. For example, the median of Jilin Province in this study was only 0.04; however, the maximum annual risk was estimated in 22.39. Similarly, the median value for Shandong province was zero, leading us to believe that there were no epidemics over the past 5 years; however, there were actually 2 years with a value of 0.58 and 0.17, respectively, revealing the existence of epidemic diseases. For those reasons, the authors preferred to use the mean to describe the central tendency of the distributions.

**Table 1:** Epidemic index of animal disease in various provinces (2010–2014).

**Province**	**2010**	**2011**	**2012**	**2013**	**2014**	**Average**
Guangxi	21.61	16.79	12.09	10.40	25.38	17.25
Ningxia	53.25	4.08	1.84	4.90	3.21	13.46
Chongqing	15.19	14.85	7.96	9.87	13.26	12.23
Qinghai	34.52	14.24	3.27	2.88	4.01	11.78
Xinjiang	45.17	0.00	0.18	2.12	3.44	10.18
Yunnan	3.01	5.41	12.13	13.15	5.87	7.91
Shanghai	10.59	3.58	3.13	15.62	4.28	7.44
Shaanxi	18.62	4.94	7.38	0.97	5.16	7.41
Gansu	7.08	3.85	14.63	5.83	4.97	7.27
Hunan	2.18	5.54	11.68	6.78	5.44	6.32
Hubei	3.16	13.89	5.85	4.88	1.78	5.91
Jiangxi	1.50	6.32	1.60	5.99	11.99	5.48
Tianjin	0.31	0.29	0.00	0.00	24.31	4.98
Guizhou	10.14	5.76	4.58	1.50	0.85	4.57
Jilin	0.09	0.04	0.00	22.39	0.00	4.50
Sichuan	1.42	2.91	3.59	4.85	4.26	3.41
Hainan	3.06	3.29	4.66	2.30	1.52	2.97
Anhui	2.47	1.83	4.85	1.29	0.82	2.25
Zhejiang	3.80	1.91	1.52	1.20	2.65	2.22
Inner Mongolia	5.69	0.15	0.02	0.00	0.00	1.17
Guangdong	3.61	1.09	0.12	0.14	0.28	1.05
Fujian	3.08	0.24	0.05	0.09	0.34	0.76
Jiangsu	0.48	0.52	0.17	1.13	0.73	0.61
Heilongjiang	0.51	0.00	1.37	0.13	0.16	0.43
Hebei	0.39	0.40	0.24	0.15	0.44	0.32
Shandong	0.00	0.00	0.00	0.58	0.17	0.15
Henan	0.22	0.00	0.00	0.00	0.00	0.04
Liaoning	0.03	0.06	0.00	0.01	0.00	0.02
Shanxi	0.00	0.00	0.03	0.00	0.00	0.01
Beijing	0.00	0.00	0.00	0.00	0.00	0.00
Xizang	0.00	0.00	0.00	0.00	0.00	0.00

### Measurements and Statistical Analysis

The outbreaks and prevalence of animal epidemic diseases are influenced by complex natural and social factors, along with interregional differences. This study used 21 regions with a higher risk index in the following panel data model:

(2) $$\begin{array}{l} Y_{it}=C+\beta_{1}A1_{it}+\beta_{2}A2_{it}+\beta_{3}A3_{it}+\beta_{4}A4_{it}+\beta_{5}B1_{it}\\ \;+\beta_{6}B2_{it}+\beta_{7}B3_{it}+\beta_{8}B4_{it}+\beta_{9}C1_{it}+\beta_{10}C2_{it}+\varepsilon_{it}\; \end{array}$$

where *Y*_*it*_ represents the regional animal epidemic index; series A, B, and C represent geographic and ecological factors, animal feeding and production levels, and animal disease prevention and control factors, respectively; *i* = 1, 2, …, 21 for 21 regions; *t* = 2010, 2011, …, 2014 for the respective years; beta subscripts 1, 2, …, 10 represent the parameters to be estimated, constant *C* is the drift term, and ε_*it*_ is the random term.

Several studies have discussed the risk factors associated with the prevalence of animal diseases, such as farm management practices (18), forest (19), soil water content (20), and water source (21). Evidence suggests that the spread of epidemic diseases is closely related to environmental factors, humans, and animals (22). Following the study of Liang et al. (23), we proposed the explanatory variables shown in Table 2.

**Table 2:** Factors influencing the epidemic risk of animal diseases.

**Factor**	**Number**	**Name**	**Unit**	**Description**	**Direction of change**
Geographic and ecological factors	1	A1 Forest coverage rate	Rate	Forestry area/acreage	+/-
	2	A2 Intensity of fertilizer	10,000 tons/1,000 ha	Fertilizer input/effective irrigation area	+
	3	A3 Sewage discharge volume	10,000 tons/1,000 ha	Sewage discharge volume/acreage	+
	4	A4 Sulfur dioxide emissions	10,000 tons / 1,000 of hectares	Sulfur dioxide emissions/acreage	+
Animal feeding and production level factor	5	B1 Rate of large-scale animal-raising households	Rate	Large-scale animal-raising households/number of households In this study, large-scale animal-raising households are those with more than 50 animals.	+/-
	6	B2 Slaughter rate	Rate	Slaughter rate of the year/breeding stock	+/-
	7	B3 Per capita animal breeding	Number of animals/per person	(breeding stock + slaughter rate of the year)/population of the year	+
	8	B4 Proportion of animal production	Rate	Pig production/regional animal husbandry output value	+/-
Animal disease prevention and control factor	9	C1 Rate of veterinary stations construction	Rate	Animal husbandry and veterinary station in villages and towns/number of township-level districts	_
	10	C2 Rate of technicians	Rate	Technician number/staff number	-

#### Geographic and Ecological Environmental Factors

The geological and ecological environmental factors that influence disease transmission mainly include the geographical environment, soil, water, and air. Specific geographic conditions (sea, river, and forest) impose certain restrictions on the transfer of infection and creating natural isolation conditions. Excessive fertilization causes a series of environmental pollution and damage, such as an imbalance in soil nutrients. Due to the high level of water consumption by pig breeding, sewage discharge will pollute the local water resources to a certain extent, thus affecting animal health. China is the world's largest coal producer, predominantly high-sulfur coal (>2.5% sulfur content), which accounts for 20–25% of the total coal reserves. In China, 84% of the total coal consumption is burned directly; during this process, a large amount of sulfur dioxide (SO_2_) is emitted. The emission of SO_2_ from coal burning accounts for more than 85% of the total SO_2_ emissions, causing serious air pollution. Therefore, this study selected the forest coverage rate, intensity of fertilizer, sewage discharge volume, and SO_2_ emissions as geographical ecological environmental factors and demonstrated the geographical and ecological environment of a region in three respects: soil, water, and air.

#### Animal Feeding and Production Levels

This study evaluated the regional animal feed production level through four indices and studied the effects of each index on animal epidemic disease risk. The first was the degree of large-scale animal-raising households, measured in terms of proportion; the higher the rate, the greater the cultivation scale. The second was animal productivity, measured using the slaughtering rate; the higher the slaughtering rate, the higher the level of animal productivity. The third was per capita animal breeding, measured in terms of breeding density; the higher the breeding density, the higher the per capita animal breeding. The fourth was the proportion of animal production, measured using the proportion of animal husbandry output; the higher the rate, the better the production efficiency.

#### Animal Epidemic Disease Prevention and Control

The existence of animal husbandry and veterinary stations in townships and their technology level affect animal epidemic prevention directly. The collection of related data is thus restricted because of its complexity. Therefore, this study evaluated the regional animal disease prevention and control management level using the ratio of animal husbandry and veterinary stations in townships and the disease prevention and control level based on the proportion of technical personnel.

## Results

The data were obtained mainly from Veterinary Bulletins, animal husbandry and veterinary yearbooks, and China's Statistical Yearbooks for 2011 to 2015. The frequency of animal epidemic diseases, actual disease prevalence, number of animal deaths, and culling data were obtained from Veterinary Bulletins from 2011 to 2015. The values of breeding stock, slaughter, animal-raising households, output of animal husbandry, regional total output of animal husbandry, number of animal husbandry and veterinary stations, and technical personnel were obtained from China's Animal Husbandry and Veterinary Yearbooks for 2011 to 2015. The forest coverage rate, fertilizer content, effective irrigation area, sewage discharge volume, emissions of SO_2_, regional total population at year-end, and the number of townships were obtained from the 2011–2015 statistical yearbooks.

### Animal Epidemic Risk Ranking

**Figure 1:**
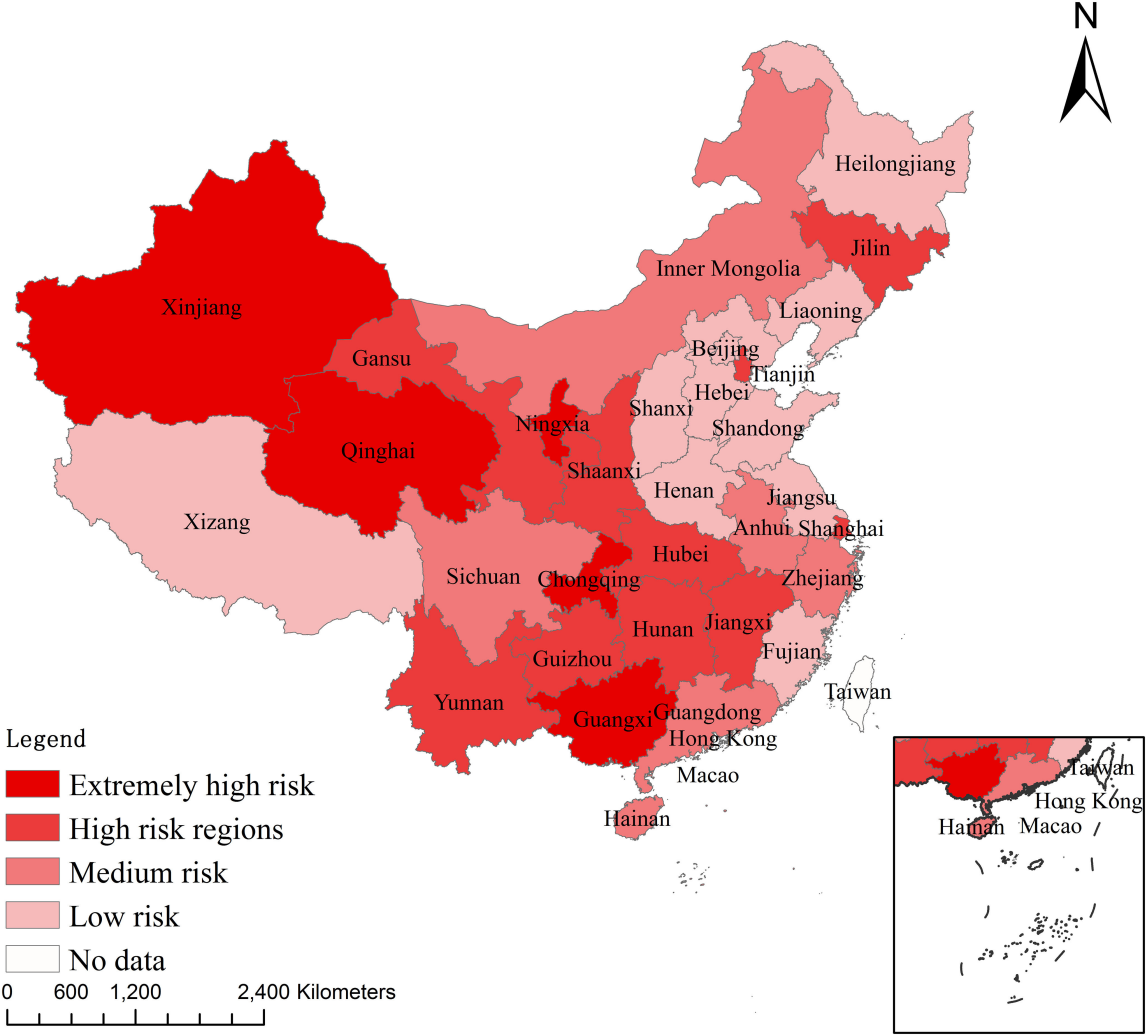
Animal epidemic risk ranking.

Using Li and Qin's method (17), we considered the median of the epidemic index of animal diseases as the threshold value. Regions with an animal epidemic index greater than the median were considered to be at high epidemic risk. Conversely, regions with a value less than the median were considered to be at less epidemic risk. The 31 provinces, municipalities, and autonomous regions in mainland China are classified into four different risk levels: extremely high risk (E ≥8), high risk ($8>\bar{\text{E}}\geq 4$), medium risk ($4>\bar{\text{E}}\geq 1$), and low risk ($1>\bar{\text{E}}\geq 0$). Therefore, Guangxi, Ningxia, Chongqing, Qinghai, and Xinjiang are classified as extremely high-risk regions; Yunnan, Shanghai, Shaanxi, Gansu, Hunan, Hubei, Jiangxi, Tianjin, Guizhou, and Jilin are classified as high-risk regions; Sichuan, Hainan, Anhui, Zhejiang, Inner Mongolia, and Guangdong are classified as medium-risk regions; and Fujian, Jiangsu, Heilongjiang, Hebei, Shandong, Henan, Liaoning, Shanxi, Beijing, and Tibet are classified as low-risk regions (Figure 1).

### Differences Between Risk Regions

**Figure 2:**
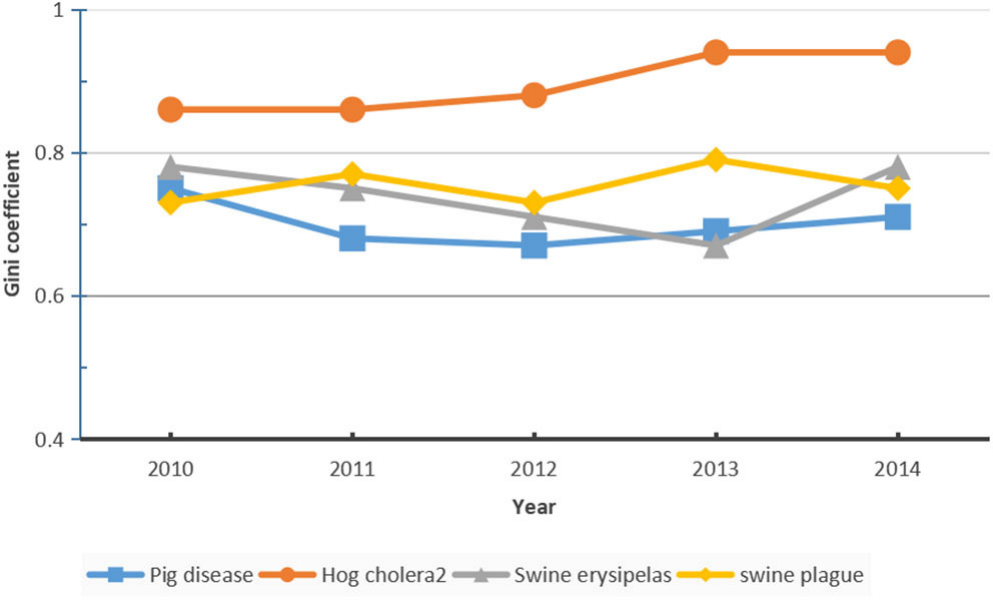
Epidemic index of animal diseases.

The Gini coefficient, which is extensively applied in the literature to evaluate various types of distribution differences (24), was adopted in this study to calculate the differences in the animal epidemic index from 2010 to 2014 across the 31 provinces, municipalities, and autonomous regions in mainland China. The Gini coefficient equation is as follows:

(3) $$\begin{array}{l} Gini=\sum_{i=1}^{n}\sum_{j=1}^{n}|y_{i}-y_{j}|/2n\left(n-1\right)Y \end{array}$$

where y_i_ stands for the animal epidemic index in region *i, n* is the number of regions, and *Y* is the average value of the animal epidemic index across the country. The Gini coefficient is a real number with values ranging from 0 (absolute equality) to 1 (absolute inequality) (25, 26). For this study, the higher the numerical value, the more significant the regional difference. As shown in Figure 2, the Gini coefficients of classical swine fever, swine erysipelas, swine plague, and the overall Gini coefficients are estimated, they showed significant interregional differences (with Gini coefficients ranging from 0.67 to 0.94) (Figure 2).

The Gini coefficient of classical swine fever was higher than that of the other two, being above 0.80 for 5 consecutive years. Therefore, among the three animal epidemic diseases, the classical swine fever epidemic showed the highest interregional differences. For overall animal epidemic diseases, the Gini coefficient of the animal epidemic index was maintained above 0.60, showing the highest value of 0.75 in 2010 and the lowest value of 0.67 in 2012, which indicated considerable interregional differences in animal epidemic risks in mainland China. The fluctuation in interregional differences was only minimal; for example, the differences decreased from 2010 to 2012 and gradually increased from 2012 to 2014.

### Regression Analysis

The panel data model was estimated using STATA, and the estimation results are shown in Table 3.

**Table 3:** Estimation of the panel data model.

**Model**	**(1)**	**(2)**	**(3)**
	**Fixed-effects model**	**Random-effects model**	**Corrected cross-section heteroscedasticity and serial correlation**
A1	−0.05821	2.64095	−0.05821
	(−0.001)	(0.252)	(−0.003)
A2	9.66272	8.06720	9.66272
	(0.084)	(0.273)	(0.180)
A3	0.87428***	−0.00366	0.87428***
	(2.872)	(−0.103)	(4.142)
A4	−0.21221***	0.02084	−0.21221***
	(−2.876)	(0.631)	(−4.276)
B1	−175.9732***	2.99862	−175.9732***
	(−2.897)	(0.283)	(−4.306)
B2	−17.01951**	−4.16416	−17.01951*
	(−2.020)	(−0.946)	(−2.017)
B3	40.61058	0.04196	40.61058*
	(1.138)	(0.010)	(1.769)
B4	12.78605	−7.84189	12.78605
	(0.274)	(−0.789)	(1.464)
C1	1.77686	8.02800	1.77686*
	(0.150)	(0.972)	(1.863)
C2	−36.31129	8.22297	−36.31129**
	(−1.210)	(0.787)	(−2.631)
_cons	17.04561	1.18208	17.04561
	(0.304)	(0.080)	(0.986)
*N*	105	105	105
r2_w	0.19272	0.00284	0.19272
F/wald	1.76663	7.65	60.57127
*P*_value (*F*)	0.0819	0.6625	0.0000
Re or Fe	Hausman test results: χ^2^(10) = 22.88, Prob = 0.0112		
Serial correlation test	*F*_(1, 20)_ = 14.995, Prob = 0.0009		
Heteroscedasticity testing between groups (FE)	χ^2^(21) = 6034.86, Prob = 0.0000		

***, **, and * represent the 1, 5, and 10% significance levels, respectively.

According to the results, the fixed-effects model *F* passed the 10% significance level test, whereas the random-effects model failed the Wald test, which indicated a non-significant linear relation of the random-effects model. Based on the Hausman test results, the fixed-effects model above the 5% significance level was superior to the random-effects model. Thus, the fixed-effects model was selected. From heteroscedasticity testing and serial correlation tests on the fixed-effects model, the XTSCC command was employed to correct the cross-section heteroscedasticity, serial correlation, and inter-block correlation problems, obtaining the following regression estimation function:

(4) $$\begin{array}{l} Y_{i}=17.0456-0.0582A1_{i}+9.6627A2_{i}+0.8743A3_{i}\\ \;-0.2122A4_{i}-175.9732B1_{i}-17.0195B2_{i}+40.6106B3_{i}\\ \;+12.7861B4_{i}+1.7769C1_{i}-36.3113C2_{i} \end{array}$$

The results showed that the forest coverage rate, fertilizer intensity, and live pig output ratio had a non-significant influence on the animal epidemic index. The remaining seven indices passed the 1, 5, or 10% levels of significance. Among the geographic and ecological factors, the sewage discharge volume per unit area had a significant positive effect on the animal epidemic index. The animals' autoimmunity would be weakened and the probability of transmission of epidemic pathogens would increase with the deterioration of the animals' living environment caused by pollution and the continuous discharge of wastewater per unit area. Another index that weighed the ecological environment condition was SO_2_ emission per unit area; the influence of SO_2_ on the animal epidemic index would theoretically be positive. However, the results were contrary to expectations, possibly because of the oxidizable and sterilizable sulfurous acid formed by SO_2_ and water, which can inhibit the transmission of animal epidemic pathogens. Evidently, an excessively high SO_2_ content in the atmosphere will inevitably expedite the formation of acid rain, which will be detrimental to the environment.

In terms of the animal feeding and production level factor, the ratio of large-scale animal-raising households negatively correlated with the animal epidemic index, implying that the larger the scale of animal-farming households, the lower the animal epidemic index. In other words, the probability of outbreaks of animal epidemic diseases in free-ranging households is higher than that in large-scale animal-raising households because of the limited funds and techniques of the former as well as their inadequate epidemic prevention facilities and conditions, as compared with large-scale animal-raising households. The ratio of large-scale animal-raising households represents the feeding model, and the slaughtering rate is used to weigh animal productivity. According to these data, the slaughtering rate negatively correlated with the animal epidemic index, as the higher the animal's productivity level, the higher the investment, benefit, and disease prevention and control awareness. As for the animal feeding and production level factor, the number of animals raised per capita positively correlated with the animal epidemic index, implying that a high breeding density would intensify the transmission of animal diseases by cross-infection.

Regarding the animal disease prevention and control factors, the rate of animal husbandry and veterinary stations positively correlated with the animal epidemic index, which was contrary to expectations, conceivably because of the low rate of animal husbandry, veterinary stations, and data acquisition, resulting in an incomplete collection of epidemic disease data and consequently in unreliable statistical results. The technical staff ratio, as an index for weighing disease prevention control and technological level, negatively correlated with the animal epidemic index. The higher the disease prevention control and technological level, the lower the epidemic index, mainly because the technical staff were able to eradicate outbreaks and the spread of epidemic diseases by prevention and surveillance, quarantine supervision, and emergency responses.

### Regional Difference Breakdown

Shapley values are widely used in the study of regional disparities. Shorrocks adopted Shapley values to calculate the contribution of explanatory variables to the income differences in the earnings function (27). Wan and Zhou improved these values to quantify the contribution of explanatory variables to the degree of inequality of explanatory variables based on regression equations (28). In the present study, the Shapley value decomposition method was applied to the breakdown of differences in animal epidemic risks across regions, through which the contribution of each determinant to the interregional differences was studied.

The factors influencing animal epidemic risk across regions comprise a set of *N* = {1, 2, …, n} influential factors. As the interregional differences in animal epidemic risk are influenced by geographic and ecological factors, animal feeding, animal production level, and disease prevention and control factors, respectively, are denoted by (Z1, Z2, and Z3) for *n* = 3. These three determinants are competing factors for the interregional differences in animal epidemic risk.

In the event of a random set S ⊆ N, S is denoted as a league of *N*, and |s| = s represents the number of elements in set *S*. As the three determinants of interregional differences in animal epidemic risks are participating factors, the non-participating influential factors are averaged in the calculations. For instance, when geographic and ecological factors are averaged, the actual observed values will be obtained from the other two factors ($\bar{Z}$1, Z2, and Z3).

The Gini coefficient is an eigenfunction of each league *S* in *N*, denoted by ν (.). When league *S* = {Z2, Z3}, the value of the eigenfunction ν (.) is ν ($\bar{\text{Z}}$1, Z2, Z3).

Consequently, the Shapley value equation can be written as follows:

(5) $$\begin{array}{l} {\phi_{i}}_{\left[v\right]}=\underset{S\subseteq n}{\sum }y_{n}\left(S\right)\left[v\left(S\right)-v\left(S-\left\{i\right\}\right)\right],\forall_{i}\subseteq N \end{array}$$

where *y*_*n*_(*S*) is the weighting factor of each league, *s* stands for the number of members in league *S*, known as the influential change factor, and [*v*(*S*)–*v*(*S*–{*i*})] can be construed as the contribution margin of *i* ⊆ *N* to league *S*. As shown in Table 4, all possible weighted means of the three determinants and, therefore, their contribution margins to and weights in the interregional difference in animal epidemic risk, are computable.

**Table 4:** Marginal contributions of influencing factors.

**Way**	**Marginal contribution**			**Weight**
	**Z_**1**_**	**Z_**2**_**	**Z_**3**_**	
1	v(Z_1_,Z_2_,Z_3_)—v(Z_1_,Z_2_,Z_3_)	v(Z_1_,Z_2_,Z_3_)—v(Z_1_,Z_2_,Z_3_)	v(Z_1_,Z_2_,Z_3_)—v(Z_1_,Z_2_,Z_3_)	$\frac{2}{6}$
2	v(Z_1_,Z_2_,Z_3_)—v(Z_1_,Z_2_,Z_3_)	v(Z_1_,Z_2_,Z_3_)—v(Z_1_,Z_2_,Z_3_)	v(Z_1_,Z_2_,Z_3_)—v(Z_1_,Z_2_,Z_3_)	$\frac{1}{6}$
3	v(Z_1_,Z_2_,Z_3_)—v(Z_1_,Z_2_,Z_3_)	v(Z_1_,Z_2_,Z_3_)—v(Z_1_,Z_2_,Z_3_)	v(Z_1_,Z_2_,Z_3_)—v(Z_1_,Z_2_,Z_3_)	$\frac{1}{6}$
4	v(Z_1_,Z_2_,Z_3_)—v(Z_1_,Z_2_,Z_3_)	v(Z_1_,Z_2_,Z_3_)—v(Z_1_,Z_2_,Z_3_)	v(Z_1_,Z_2_,Z_3_)—v(Z_1_,Z_2_,Z_3_)	$\frac{2}{6}$

The contribution degree can be expressed as ϕ_*i*_/(ϕ_1_ + ϕ_2_ + ϕ_3_), where *i* = 1, 2, 3. The next step was decomposing the regional difference manually using Microsoft Excel 2010, as shown in Table 5. Owing to the effect of model residuals and the existence of irresoluble parts in the models, the results from the decomposition represent the relative degrees of contribution of the three model-based determinants.

**Table 5:** Decomposition of interregional differences in animal disease prevalence.

**Year**	**Geographic and ecological factor**		**Animal feeding and production level factor**		**Animal disease prevention and control factor**	
	**Contribution (%)**	**Ranking**	**Contribution (%)**	**Ranking**	**Contribution (%)**	**Ranking**
2010	33.13	2	40.63	1	26.24	3
2011	29.11	2	43.04	1	27.85	3
2012	29.75	2	42.41	1	27.84	3
2013	29.32	3	41.35	1	29.33	2
2014	29.91	3	39.53	1	30.56	2
Mean	30.25	2	41.39	1	28.36	3

The results show that interregional differences in animal feeding and production contributed most to the interregional differences in animal epidemic risk from 2010 to 2014, with the highest contribution in 2011, at 43.04%, and the lowest contribution in 2014, at 39.53%. The average annual contribution was 41.39%.

Animal disease prevention and control had an average degree of contribution of 28.36% and ranked third and second during 2010–2012 and 2013–2014, respectively. The differences in disease prevention and control in various regions are mainly reflected in epidemic disease prevention and surveillance, quarantine supervision, and emergency response. The introduction of additional veterinary stations and technical staff could effectively minimize outbreaks and transmission of animal epidemic diseases, thus improving regional disease prevention and control levels.

## Conclusions

According to the findings of this study, the prevalence of classical swine fever, swine erysipelas, and swine plague in different regions in mainland China from 2010 to 2014 was characterized by significant differences in outbreak frequency, number of animals affected by the disease, number of deaths, and number of animals slaughtered. The epidemic situation in each region was estimated by applying the epidemic index formula, for which the 31 provinces, municipalities, and autonomous regions in mainland China were divided into extremely high-, high-, medium-, and low-risk regions using the animal epidemic index. Moreover, the Gini coefficient of the index remained above 0.60 for these 5 consecutive years, signifying the existence of tremendous interregional differences in animal epidemic risks in mainland China. Next, the causes of such interregional differences were decomposed using the Shapley value method. The results showed that 41.39% of the interregional differences arose from differences in the animal feeding and production levels, whereas 30.25 and 28.36% of the differences arose from differences in geographic and ecological factors, and animal disease prevention and control factors, respectively.

Overall, the regional animal feeding and production levels play an important role in the interregional differences in animal epidemic risk. The improvement in regional animal production levels and shifting from free-ranging to large-scale farming are conducive to epidemic disease prevention and control, while breeding density should be controlled to inhibit the spread of diseases. In terms of geographic and ecological factors, inadequate sewage discharge regulation increases the chances of pathogen transmission, whereas, in terms of disease prevention and control, introducing more livestock veterinary staff is important.

It is suggested that a regionalized administration system covering detailed animal epidemic prevention and control strategies by disease species, region, and phase should be established and improved to create better conditions for regionalized animal disease management in view of interregional differences in animal epidemic risk. The geographic and environmental constraints in different regions should also be considered when developing breeding programs and shifting the breeding mode from free-ranging to large-scale farming by introducing appropriate policies. On this basis, the role of technical staff in animal disease prevention and control should also be emphasized to improve regional epidemic disease prevention and supervision, quarantine, and emergency responses. Limitations of this study are as following: firstly, this study mainly focuses on environmental factors that cause epidemic disease, while host factors (such breed, age, and immunity) and agent factors (causative agents, its strains, and pathogenicity) have not been included. Secondly, although some of the variables selected in this study have reflected the biosecurity status of the farm to a certain extent, however, there is a lack of statistical data on farm biosecurity level variables (such as the protective conditions of the feedlots, and the distance between farms). Thirdly, the data used in this study are mainly based on the China statistical yearbook, which has the limitation of data acquisition. Nevertheless, there are more factors which may contribute to the regional differences in epidemics, such as under-reporting of diseases (endemic), obtaining data from passive surveillance, trading of live animals, and educating farmers about risk awareness. Future research directions could take a more comprehensive consideration and work on the above limitations.

## Data Availability Statement

The datasets generated for this study are available on request to the corresponding author.

## Author Contributions

YL: conceptualization, investigation, project administration, methodology, writing-review and funding acquisition. LS: formal analysis and editing. WZ: formal analysis, methodology, writing-review, and funding acquisition. QS: investigation and data curation. All authors contributed to the article and approved the submitted version.

## Conflict of Interest

The authors declare that the research was conducted in the absence of any commercial or financial relationships that could be construed as a potential conflict of interest.
